# How to promote the hierarchical diagnosis and treatment system: A tripartite evolutionary game theory perspective

**DOI:** 10.3389/fpsyg.2022.1081562

**Published:** 2023-01-05

**Authors:** Chunhai Tao, Xi Chen, Wenji Zheng, Zehao Zhang, Ruoyan Tao, Rui Deng, Qizhe Xiong

**Affiliations:** ^1^School of Statistics, Jiangxi University of Finance and Economics, Nanchang, China; ^2^School of Economics and Management, Nanchang University, Nanchang, China; ^3^School of Liberal Arts, Macau University of Science and Technology, Taipa, Macau SAR, China

**Keywords:** hierarchical diagnosis and treatment, evolutionary game theory, prospect theory, stochastic disturbance, medical cultures

## Abstract

Due to the disorderly access to medical care and inefficient use of health resources, the advancement of the hierarchical diagnosis and treatment is more valued in promoting health system reform. Hence, this article integrates prospect theory into an evolutionary game model of the local government health departments, the medical institutions, and the patients in the system promotion of the hierarchical diagnosis and treatment. The simulation shows the specific influencing mechanism of the psychological perceived value of game subjects. Then by introducing the stochastic evolutionary game model, the system promotion under different medical cultures is also discussed in detail. The results indicate that for local government health departments, the amount and duration of financial subsidies are the key factors influencing the game system’s evolution. For medical institutions, participating in the hierarchical diagnosis and treatment system is relatively beneficial. For patients, the recovery rate in primary hospitals matters more than the cost of treatment. Changes in the risk sensitivity coefficient will cause the equilibrium of the game system to change. However, changes in the loss avoidance factor do not change the equilibrium and only have an impact on the speed of convergence. With the health departments’ intervention, patients in rural medical culture are more inclined to support the hierarchical diagnosis and treatment system than those in urban or town medical culture. Therefore, in order to promote the hierarchical diagnosis and treatment system, this article recommends that more attention should be paid to the regulatory role of health departments and the participation improvement of medical institutions and patients.

## Introduction

Since the implementation of reform and opening up, China’s public health system has been gradually improved and the scale of medical services has been further expanded (Yip et al., 2019). However, at the same time, the construction and improvement of the medical service system have faced developmental difficulties, which leads to hardship in implementing a national hierarchical medical treatment system in China and other developing countries (Xie et al., 2022). First, the irrational distribution of medical services at all levels of medical institutions has led to the overcrowding of secondary and tertiary hospitals and the underutilization of resources in primary-level health care institutions (Liu et al., 2017). Second, due to the “inverted pyramid” structure of urban and rural health care resource allocation (Yi et al., 2020), the problems of disorderly access to medical care and inefficient utilization of health resources as a whole have begun to emerge (Sun and Luo, 2017), which not only affects the operational efficiency of the medical service system but also increases the medical expenses of patients, thus increasing their financial burden and the pressure on health insurance funds (Li et al., 2021). In response to these problems, China State Council has implemented a hierarchical diagnosis and treatment system (HDTS), the connotation of which is that, according to the priority of the patient’s disease and the difficulty of treatment, medical institutions of different levels are responsible for the treatment of different diseases, while timely and convenient two-way referrals are made according to the changes in the condition so as to establish a scientific and orderly treatment and to ensure that patients receive appropriate treatment (Liang et al., 2019). Thus, promoting the construction of HDTS can guide patients to seek medical treatment in an orderly manner, to continuously optimize the allocation of health resources, to alleviate the contradiction between supply and demand of medical services, and to control the unreasonable rise of medical costs so as to effectively solve the problem of difficult and expensive medical care for residents (Huo et al., 2019).

For the exploration in the field of the public health system, researchers mainly conducted empirical analyses from the perspective of the integrated implementation of primary-level care as well as a referral system, which mainly connotes the division of labor between general practitioners and specialists in cooperation (Stirbu et al., 2011), and some scholars also studied the integration of health services (Li et al., 2011), and through in-depth studies, researchers found that it is essential to achieve consistency and collaboration among various departments within the health system (Gao et al., 2020). Further integration of existing health care resources to improve the utilization of health care resources is the main point of discussion at present, and relatively few relevant studies have been conducted considering the specific problems in the Chinese health care environment and the decision-making of participants in HDTS.

Along with rapid economic growth, China has achieved a relatively rapid increase in health care expenditure as a proportion of GDP (Jakovljevic et al., 2022), but there is still room for improvement compared to developed countries such as the United States, Canada, and France (Lopreite and Zhu, 2020). At present, the problem of scarcity of medical resources in China has been alleviated, but the distribution of medical resources, especially high-quality resources, still needs to be addressed (Liu et al., 2008). In addition, under the influence of the traditional concept of medical care, Chinese patients tend to go directly to tertiary hospitals for the first consultation and are often reluctant to consult primary medical institutions due to distrust of treatment effectiveness and dissatisfaction with medical services (Zhang et al., 2018).

The establishment and implementation of HDTS is a key task needed to be accomplished to realize the reform of the medical and health system under the unique medical culture of China. The effective implementation of this system cannot be achieved without the cooperation of government health departments, medical institutions at all levels, and patients (Liu et al., 2008). Therefore, studying the internal coordination and decision-making of HDTS can further optimize the operating mechanism of the system (Ma et al., 2008), realize the synergy of multiple participants, and ultimately build up a new pattern of medical care which enhances the sense of wellbeing in seeking medical care for Chinese residents.

Existing studies on the implementation of HDTS center on the following aspects: (1) suggestions for the development of primary care based on modeling and empirical evidence (Woodruff, 2022), (2) the regulatory role of health insurance (Hennig-Schmidt et al., 2011), and (3) referrals and cooperation between general practitioners and specialists (Ringberg et al., 2014). Recently, factors such as government subsidies and the efficiency of medical services have been the focus of discussion.

Existing literature on HDTS is mainly about two-sided games and seldom considers constructing a tripartite game model. Most relevant studies had not taken into account the limited rationality of decision-makers and the influence of external unstable factors on HDTS. Therefore, our research analyzed the influence of risk sensitivity and loss aversion of government, hospitals, and patients on the optimal results based on prospect theory and also present the impact on decision-makers under the influence of psychological factors through simulations. Further, we analyzed the random interference of uncertainties such as changes in the operating environment of the health care system and unexpected personal situations of patients to make the analysis results more related to the actual situations in China. The associations of psychological factors and promoting HDTS are further explored when our study represents one of the first attempts to introduce prospect theory and stochastic disturbance to optimize the game model. It will be helpful to enrich the existing relevant research and provide a basis for further in-depth studies.

## Literature review

### Hierarchical diagnosis and treatment and the decision-making of each subject

Medical services for common diseases are in great demand, and these kinds of diseases should be addressed in primary-level care institutions due to the regularity (Yang et al., 2021); the difficult and serious diseases, which have a lower incidence and are more difficult to treat than common diseases, need to be treated in secondary and tertiary hospitals with high quality medical resources (Shen et al., 2019). However, the improvement in the standard of living and accessibility of medical services has led to a tendency for residents to choose secondary and tertiary hospitals for the first consultation after suffering from diseases, making tertiary hospitals, which are supposed to mainly treat difficult and critical diseases, overcrowded (Li et al., 2020). The “siphon effect” of tertiary hospitals is serious (Wu Q. et al., 2022), driving the number of patients to decrease rapidly in primary-level care institutions (Zeng et al., 2020), which is contrary to the goal of promoting HDTS, and this problem needs to be solved urgently. This phenomenon is related to patients’ medical concepts and habits (Chandra and Staiger, 2007), primary care service capacity (Jian et al., 2019), public health policies (Maxwell et al., 2008), and the cooperation mechanism of medical institutions at all levels (Li et al., 2017). Therefore, the formulation and promotion of HDTS require the coordination of local government health departments, medical institutions at all levels, and patients (Jian et al., 2019).

First, the government health department should consider the comprehensive background to formulate precise and effective policies, while supervision is needed at all levels to put various policies into practice (Huang, 2014). Along with the accelerated urbanization process and the implementation of the rural revitalization strategy (Meng et al., 2012), the government can adjust health expenditure policies flexibly and give full play to the role of the market (Song et al., 2020). In the specific implementation process, the government can require subordinate departments and medical institutions to participate in optimizing the medical service environment through incentive and punishment mechanisms (Whetten et al., 2006). Second, active cooperation between medical institutions helps build an integrated health care system (Stock, 2015), which can achieve effective utilization of medical resources through information sharing (Caine and Hanania, 2013). The rational layout and orderly sinking of medical resources can reduce the “siphon effect” of tertiary hospitals (Wu X. et al., 2022). Finally, patients tend to judge the quality of hospital services based on whether they can provide the facility to treat major diseases or perform difficult surgeries (Niu et al., 2019), which is the fundamental reason why community-level medical institutions are not trusted by patients (Sanders et al., 2015). Choosing tertiary hospitals for the first consultation without complying with HDTS not only increases medical expenses (Dong et al., 2019) but also results in additional waiting costs for patients (Yin et al., 2018). In addition to the waiting time, the treatment rate and the reimbursement rate of medical insurance will also affect the patient’s decision to seek medical treatment (Klest et al., 2016). The evolutionary game model is used to deduce the evolutionary conditions and evolutionary process of the participants in HDTS and then to analyze the main influencing factors of the participants in the decision-making process. Related studies portrayed the evolutionary game paths of decision-making subjects in public health emergencies (Fan et al., 2021), and some scholars analyzed the optimal strategies of three entities, namely, system providers, hospitals, and the government, in the context of mobile medical systems, emphasizing the positive role of government regulation (Yuan et al., 2022).

### Application of prospect theory in the evolutionary game

Most of the game models are constructed based on the premise of objective gains and losses, and the finite rationality of the decision subjects is not sufficiently considered. However, in the actual analysis, the perceived gain and loss of each subject in the game model will have a more significant impact on their behavior, which means that the game subject is essentially finite rational, so the introduction of prospect theory can make the model used in the study more suitable for the actual situation. Prospect theory, jointly proposed by Daniel Kahneman and Amos Tversky, belongs to a branch of cognitive decision theory within the descriptive research paradigm in political psychology, which is a combination of psychology and economics and is often used to study subjects’ decisions under uncertainties in social sciences. Prospect theory considers psychological preferences and quantifies them in people’s behavioral decisions. Most of the current studies used the parameters of the value function and probability weight function proposed by Kahneman and Tversky, specifying the exact values of the different parameters. According to prospect theory, decision-makers’ perceptions of losses and benefits are asymmetric. The primary motivation of decision-makers is loss avoidance rather than benefit pursuit, so decision-makers tend to be risk-seeking when facing losses and risk-averse when facing benefits, and thus, preferences are probabilistically non-linear (Barberis, 2013). Prospect theory suggests that subjects in a decision-making position place more importance on the amount of change in benefits than on the final quantity (Grinblatt and Han, 2005).

Currently, prospect theory has been widely used in evolutionary game models to study the influence of psychological factors of subjects on decision-making, and some scholars used prospect theory to analyze the influence of risk factors on addictive behaviors (Cabedo-Peris et al., 2022). Prospect theory also has a wide range of applications in health care research, and one study based on prospect theory has contributed a new methodological approach to the ongoing efforts toward evaluating public health surveillance, revealing that patients tend to underestimate benefits and overestimate risks (Attema et al., 2019). Also, studies argued that prospect theory can optimize disease identification methods (Castiglione et al., 2017). Related studies considered the impact of global awareness and risk aversion factors of decision-makers on the evolution of the game system under prospect theory (Alfinito et al., 2018), and some scholars argued that the recommendation of consumer subjects can bring subsequent trust gains and profitability to operators (Kumar et al., 2010). A study was conducted to analyze the behavioral decisions of insured patients, providers, technology companies, and policymakers in the context of technological advances in conjunction with prospect theory (Khan et al., 2022).

### Stochastic evolutionary game model

The classical evolutionary game theory treats each game party as a community rather than an individual, and each member of the community may not be able to adopt the strategy that maximizes its own gain at the initial moment but gradually adjust its own strategy in the process of the continuous game so that its own gain is constantly optimized.

The disadvantage of this approach is that it does not take into account the fact that the game parties cannot always maintain rationality in the process of strategy adjustment. Uncertainties, such as the influence of factors such as difficulties in obtaining information and fluctuations in gains, cannot be reflected by the traditional evolutionary game model (also called the deterministic evolutionary game model) (Ajmone Marsan et al., 2016). Thus, researchers constructed a stochastic evolutionary game model. Different from the concept of evolutionary stable strategy in the deterministic evolutionary game model, researchers proposed the concept of stochastically stable strategy (SSS) for stochastic mutation stability. The biggest difference between stochastic and deterministic evolutionary games is that the former takes into account the complexity and variability of the real situation, and instead of simply writing the law of probability change over time as a deterministic formula, a stochastic perturbation term is introduced.

Under the influence of external random perturbations, individual judgments can diverge, leading to fluctuations in group strategy choice. Most of the current studies on healthcare services ignore the existence of implicit inertia in seeking medical care due to long-term urban-rural differences (Bywaters et al., 2019). Some studies discussed the influence of patients’ socioeconomic background on the choice of healthcare providers (Rees et al., 2022). Studies showed that patients’ situational decisions are influenced by the subjective judgments of family members (Chang and Basnyat, 2015), whereas other scholars argued for a relationship between food culture and health perceptions (Lee, 2004). Therefore, there is good reason to infer that the development gap between urban and rural areas brings about different cultures that influence healthcare seeking. In conclusion, stochastic perturbations such as institutional rationality and social culture can influence patients’ decisions to seek medical care, and it is not advisable to ignore the long-term role of medical cultures when studying medical problems.

## Modeling

### Assumptions and parameters

Hypothesis 1: In the process of promoting HDTS, the game subjects are local government health departments, medical institutions, and patients, and all of them are finite and rational. Strategy space for each player can adopt one of the two strategies: to be in favor of HDTS, labeled as cooperators (C), or to not in be favor of HDTS, labeled as defectors (D). The fraction of cooperators in local government health departments, medical institutions, and patients are, respectively, remarked with *x*, *y*, *z* (*x*, *y*, *z*∈[0,1]). Accordingly, the fraction of defectors is respectively remarked with 1-*x*, 1-*y*, and 1-*z.*

Hypothesis 2: Health departments in the local government actively support HDTS in two ways. The local health committee organizes professionals to improve the relevant medical regulations and ensures that medical institutions actively participate in the HDTS to maintain a high referral efficiency (Dresser and Frader, 2009) and therefore need to give financial subsidies *S* to medical institutions. Taking into account that patients adhering to HDTS will often consult primary-level hospitals for medical examination and treatment to prevent diseases from deteriorating, the local medical insurance department needs to increase the reimbursement ratio of primary-level hospitals by θ and θ ∈ (0,1) (Kessler et al., 2004). When patients cooperate with the process of primary care and two-way referral, medical resources are allocated more efficiently, creating social benefits *B*_*1*_. If patients still choose secondary and tertiary hospitals for the first consultation, the medical resources in Grade I hospitals are forced to divert to Grade II and Grade III hospitals, thus the local government health department suffers loss *L*. Here, we introduce supervision by higher authorities (Dresser and Frader, 2009), such as the National Health Commission and National Health Insurance Administration. If they find out patients do not comply with HDTS and local government health departments do not perform the above functions, the local department will be punished *P*.

Hypothesis 3: When medical institutions are cooperators in HDTS, a referral form is required and the receiving hospital needs to register temporarily, so the additional cost borne by the whole medical system is *C*_3_.

When medical institutions are defectors in HDTS, if patients cooperate in HDTS, the pressure on the admission of secondary and tertiary hospitals is appropriately relieved, and the additional cost to be borne by the medical system is *C*_*1*_; if patients do not cooperate, primary medical resources are not fully utilized, and because there are not enough reservations for registration, temporary referrals will make the operation of the medical system more burdensome, and the additional cost to be borne by the medical system is *C*_*2*_, i.e., *C*_2_ > *C*_1_ > *C*_3_.

Hypothesis 4: When patients are cooperators in HDTS, they choose Grade I hospitals for the first consultation since primary care institutions receive more patients, thus medical resources are tilted to the grassroots. The overall utilization rate of medical resources such as drugs and machinery is increased, and the medical service system gains *I*_*1*_; when patients are defectors in HDTS, the medical system still gains *I*_*2*_, i.e., *I*_1_ > *I*_2_.

Hypothesis 5: Patients’ treatment cost in primary care is *E*_*1*_ and the average treatment cost in secondary and tertiary hospitals is *E*_*2*_, so there is *E*_1_ < *E*_2_. Patients will first go to primary care institutions when they are cooperators in HDTS. We define the recovery rate after initial treatment in primary care is λ, λ ∈ (0,1); if patients are observed as ones who need an upward referral and the health care system actively implements referral measures such as the adequate provision of reservations for registration, the proportion of patients cost savings is ω, ω ∈ (0,1). Patients’ health benefit of recovery is *B*_2_.

Hypothesis 6: The choice of strategy made by the three parties is based on the perceived value of the gain and loss of the strategy rather than the real value of the gain and loss of the strategy, which is consistent with the prospect theory. The perceived value can be measured by the prospect value *V*, which is jointly determined by the value function ν(Δη_*i*_) and the weight function π(*p*_*i*_), as shown by Eq. 1.


(1) $$\left\{ \begin{array}{c} V=\sum_{i}\pi \,\left(p_{i}\right)\,\nu \,\left(\Delta \,\eta_{i}\right)\\ \nu \,\left(\Delta \,\eta_{i}\right)=\left\{ \begin{array}{c} {\left(\Delta \eta \right)}^{\alpha }\;\left(\Delta \eta_{i}\geq 0\right)\\ -\beta {\left(-\Delta \eta_{i}\right)}^{\alpha }\left(\Delta \eta_{i}<0\right) \end{array}\right. \end{array}\right.$$

In Eq. 1, *p*_*i*_ is the probability of occurrence of the event *i*. The weight function π(*p*_*i*_) has the following characteristics: π(0) = 0, π(1) = 1; π(*p*_*i*_) > *p*_*i*_ when *p*_*i*_ is small, π(*p*_*i*_) < *p*_*i*_ when *p*_*i*_ is large. These characteristics indicate that people tend to overestimate low-probability events and underestimate high-probability events, which is consistent with the state of decision-making behavior of finite rational game subjects under uncertainties. Δη_*i*_ is the difference between the game subject’s perception of the actual value of the event *i* and the value of the reference point. Influenced by the situation effect, the choice of the reference point depends on the decision maker’s subjective feelings and desired choice. The subject’s psychological perception of the decision behavior is gain when Δη_*i*_≥0 and loss when Δη_*i*_ < 0. The value function ν(Δη_*i*_) is characterized by the preference for gain and aversion of loss, which is reflected by the parameters α and *β*. α is the risk sensitivity coefficient (0 < α < 1), which indicates the degree of marginal diminution of the perceived gain and loss value of the game subject. The larger its value, the greater the marginal diminution of the perceived value. *β* is the loss avoidance factor (*β* ≥ 1). The higher its value, the greater the sensitivity of the game subject to losses. The sensitivity of the value function at Δη_*i*_ < 0 is stronger than that at Δη_*i*_ > 0, which means the value curve is steeper in the loss interval than in the gain interval.

According to the prospect theory, there is no deviation between the perceived value and the actual utility of the determining gains and losses. Only when participants are uncertain about gains and losses can psychological perceived utility be generated. In this study, the value of the reference point is set to 0. *S*, θ*E*_1_, and ω*E*_2_ have a value-perception feature, which needs to be represented by the value function: ν(*S*) = −*βS*^α^ when health departments provide subsidies and ν′(*S*) = *S*^α^ when medical institutions receive subsidies, ν(θ*E*_1_) = −*β*(θ*E*_1_)^α^ when health departments pay the additional health insurance reimbursement and ν′(θ*E*_1_) = (θ*E*_1_)^α^ when patients benefit from it, and ν(ω*E*_2_) = (ω*E*_2_)^α^ when patients and medical institutions are both cooperators in HDTS. The payoff matrix is constructed and shown in Table 1 (Bandyopadhyay et al., 2015).

**TABLE 1:** Payoff matrix of the tripartite evolutionary game in the promotion of HDTS.

Strategies			Payoffs		
G	H	P	G	H	P
C	C	C	*B*_1_ + ν(*S*) + ν(θ*E*_1_)	*I*_1_−*C*_3_ + ν′(*S*)	$B_{2}-E_{1}+\nu '(\theta E_{1})-(1-\lambda )E_{2}+(1-\lambda )\nu (\omega E_{2})$
C	C	D	ν(*S*)−*L*	*I*_2_−*C*_3_ + ν′(*S*)	*B*_2_−*E*_2_
C	D	C	*B*_1_ + ν(θ*E*_1_)	*I*_1_−*C*_1_	*B*_2_−*E*_1_ + ν′(θ*E*_1_)−(1−λ)*E*_2_
C	D	D	−*L*	*I*_2_−*C*_2_	*B*_2_−*E*_2_
D	C	C	*B*_*1*_	*I*_1_−*C*_3_	$B_{2}-E_{1}-(1-\lambda )E_{2}+(1-\lambda )\nu (\omega E_{2})$
D	C	D	−*L*−*P*	*I*_2_−*C*_3_	*B*_2_−*E*_2_
D	D	C	*B*_*1*_	*I*_1_−*C*_1_	*B*_2_−*E*_1_−(1−λ)*E*_2_
D	D	D	−*L*−*P*	*I*_2_−*C*_2_	*B*_2_−*E*_2_

G, the local government health departments; H, medical institutions (hospitals); P, patients.

### Analysis of evolutionarily stable strategies for replicator dynamic equations

(1) Local government health departments

Local government health departments’ expected benefits *U*_*1C*_ as cooperators, *U*_*1D*_ as defectors, and the average expected benefits *U*_*1*_ are as follows:


(2) $$\left\{ \begin{array}{c} U_{1\,C}=y\,z\,\left[B_{1}+\nu \,\left(S\right)+\nu \,\left(\theta \,E_{1}\right)\right]+y\,\left(1-z\right)\,\left[\nu \,\left(S\right)-L\right]\\ +\left(1-y\right)\,z\,\left[B_{1}+\nu \,\left(\theta \,E_{1}\right)\right]+\left(1-y\right)\,\left(1-z\right)\,\left(-L\right)\\ U_{1\,D}=y\,z\,B_{1}+y\,\left(1-z\right)\,\left(-L-P\right)\\ +\left(1-y\right)\,z\,B_{1}+\left(1-y\right)\,\left(1-z\right)\,\left(-L-P\right)\\ U_{1}=x\,U_{1\,C}+\left(1-x\right)\,U_{1\,D} \end{array}\right.$$

Therefore, the replicator dynamic equation for local government health departments is


(3) $$F(x)=\frac{dx}{dt}=x(U_{1C}-U_{1})=x(1-x)[z\nu (\text{θ}E_{1})+y\nu (S)+(1-z)P]$$

Taking the first-order derivative of *F*(*x*) yields:


(4) $$\frac{d\,\left(F\,\left(x\right)\right)}{d\,x}=\left(1-2\,x\right)\,\left[z\,\nu \,\left(\theta \,E_{1}\right)+y\,\nu \,\left(S\right)+\left(1-z\right)\,P\right]$$

Thus, all levels are in a steady state when *y* = −[*z*ν(θ*E*_1_) + (1−*z*)*P*]/ν(*S*) = y^*^, x^*^ = 1 is the evolutionarily stable strategy (ESS) when *y* < y^*^, x^*^ = 0 is the ESS when *y* > y^*^.

**FIGURE 1:**
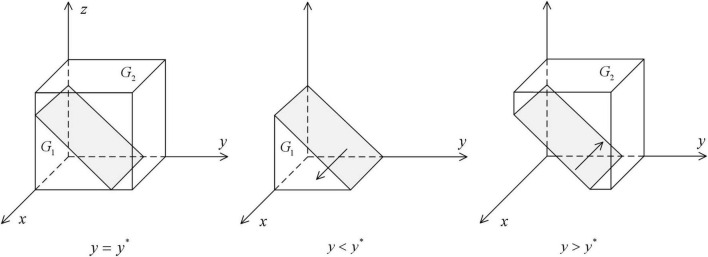
Impact of changes in the probability of strategy of medical institutions on the ESS of the local government health departments.

As shown in Figure 1, the volume *G*_*1*_ represents the probability that the local government health department cooperates in HDTS. The volume *G*_*2*_ represents the probability that they defect in HDTS. These are calculated as


$$V_{G_{1}}=\frac{P^{2}}{2\,S\,\left[P+\nu \,\left(\theta \,E_{1}\right)\right]}$$

$$V_{G_{2}}=\frac{2\,S\,\left(P+v\,\left(\theta \,E_{1}\right)\right)-P^{2}}{2\,S\,\left(P+\nu \,\left(\theta \,E_{1}\right)\right)}$$

**Corollary 1:** The probability of the local government’s health departments cooperating in HDTS decreases with the increase in the probability that medical institutions and patients cooperate with the system; at the same time, it increases with the increase in penalties from higher departments and decreases with the increase in financial subsidies to medical institutions and additional medical insurance reimbursements to primary care institutions.

(2) Medical institutions

Medical institutions’ expected benefits *U*_*2C*_ as cooperators, *U*_*2D*_ as defectors, and the average expected benefits *U*_*2*_ are


(5) $$\left\{ \begin{array}{c} U_{2\,C}=x\,z\,\left[I_{1}-C_{3}+\nu^{\prime }\,\left(S\right)\right]+x\,\left(1-z\right)\,\left[I_{2}-C_{3}+\nu^{\prime }\,\left(S\right)\right]\\ +\left(1-x\right)\,z\,\left(I_{1}-C_{3}\right)+\left(1-x\right)\,\left(1-z\right)\,\left(I_{2}-C_{3}\right)\\ U_{2\,D}=x\,z\,\left(I_{1}-C_{1}\right)+x\,\left(1-z\right)\,\left(I_{2}-C_{2}\right)\\ +\left(1-x\right)\,z\,\left(I_{1}-C_{1}\right)+\left(1-x\right)\,\left(1-z\right)\,\left(I_{2}-C_{2}\right)\\ U_{2}=y\,U_{2\,C}+\left(1-y\right)\,U_{2\,D} \end{array}\right.$$

Therefore, the replicator dynamic equation for medical institutions is as follows:


(6) $$F(y)=\frac{dy}{dt}=y(E_{2C}-E_{2})=y(1-y)[z(C_{1}-C_{2})+x\nu '(S)-C_{3}+C_{2}]$$

Taking the first-order derivative of *F*(*y*) yields:


(7) $$\frac{d\,\left(F\,\left(y\right)\right)}{d\,y}=\left(1-2\,y\right)\,\left[z\,\left(C_{1}-C_{2}\right)+x\,\nu^{\prime }\,\left(S\right)-C_{3}+C_{2}\right]$$

Thus, all levels are in a steady state when *z* = [*C*_3_−*C*_2_−*x*ν′(*S*)]/(*C*_1_−*C*_2_) = z^*^, y^*^ = 0 is the ESS when *z* < z^*^, y^*^ = 1 is the ESS when *z* > z^*^.

**FIGURE 2:**
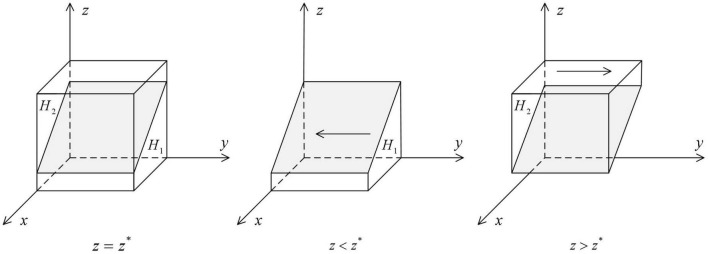
Impact of changes in the probability of strategy of patients on the ESS of medical institutions.

As shown in Figure 2, the volume of *H*_*1*_ represents the probability that medical institutions defect in HDTS, and the volume of *H*_*2*_ represents the probability that they cooperate in HDTS. These are calculated as


$$V_{H_{1}}=\frac{2\,\left(C_{3}-C_{2}\right)-\nu^{\prime }\,\left(S\right)}{2\,\left(C_{1}-C_{2}\right)}$$

$$V_{H_{2}}=\frac{2\,\left(C_{1}-C_{3}\right)+\nu^{\prime }\,\left(S\right)}{2\,\left(C_{1}-C_{2}\right)}$$

**Corollary 2:** The probability of medical institutions cooperating in HDTS increases with the increase in the probability that the local government health departments and patients cooperate with the system; at the same time, it decreases with the increase of additional costs incurred due to HDTS and increases with the increase of financial subsidies received or costs to be borne if they do not participate in the system.

(3) Patients

Patients’ expected benefits *U*_*3C*_ as cooperators, *U*_*3D*_ as defectors, and the average expected benefits *U*_*3*_ are


(8) $$\left\{ \begin{array}{c} U_{3\,C}=x\,y\,\left[B_{2}-E_{1}+\nu^{\prime }\,\left(\theta \,E_{1}\right)-\left(1-\lambda \right)\,E_{2}+\left(1-\lambda \right)\,\nu \,\left(\omega \,E_{2}\right)\right]\\ +x\,\left(1-y\right)\,\left[B_{2}-E_{1}+\nu^{\prime }\,\left(\theta \,E_{1}\right)-\left(1-\lambda \right)\,E_{2}\right]\\ +\left(1-x\right)\,y\,\left[B_{2}-E_{1}-\left(1-\lambda \right)\,E_{2}+\left(1-\lambda \right)\,\nu \,\left(\omega \,E_{2}\right)\right]\\ +\left(1-x\right)\,\left(1-y\right)\,\left[B_{2}-E_{1}-\left(1-\lambda \right)\,E_{2}\right]\\ U_{3\,D}=x\,y\,\left(B_{2}-E_{2}\right)+x\,\left(1-y\right)\,\left(B_{2}-E_{2}\right)\\ +\left(1-x\right)\,y\,\left(B_{2}-E_{2}\right)+\left(1-x\right)\,\left(1-y\right)\,\left(B_{2}-E_{2}\right)\\ U_{3}=z\,U_{3\,C}+\left(1-z\right)\,U_{3\,D} \end{array}\right.$$

Therefore, the replicator dynamic equation for patients is as follows:


(9) $$F\,\left(z\right)=\frac{d\,z}{d\,t}=z\,\left(U_{3\,C}-U_{3}\right)=z\,\left(1-z\right)\,\left[y\,\left(1-\lambda \right)\,\nu \,\left(\omega \,E_{2}\right)+x\,\nu^{\prime }\,\left(\theta \,E_{1}\right)+\lambda \,E_{2}-E_{1}\right]$$

Taking the first-order derivative of *F*(*z*) yields


(10) $$\frac{d\,\left(F\,\left(z\right)\right)}{d\,z}=\left(1-2\,z\right)\,\left[y\,\left(1-\lambda \right)\,\nu \,\left(\omega \,E_{2}\right)+x\,\nu^{\prime }\,\left(\theta \,E_{1}\right)+\lambda \,E_{2}-E_{1}\right]$$

All levels are in a steady state when *x* = [*E*_1_−λ*E*_2_−*y*(1−λ)ν(ω*E*_2_)]/ν′(θ*E*_1_) = x^*^, z^*^ = 0 is the ESS when *x* < x*, z* = 1 is the ESS when *x* > x*.

**FIGURE 3:**
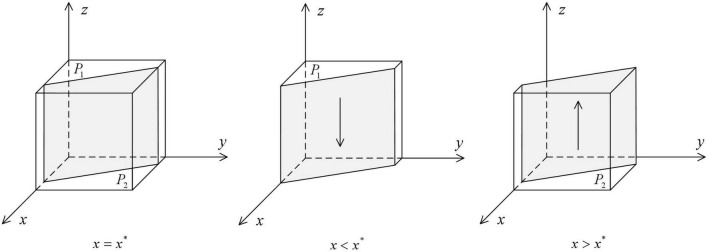
Impact of changes in the probability of strategy of local government health departments on the ESS of patients.

As shown in Figure 3, the volume of *P*_*1*_ represents the probability that patients defect in HDTS, and the volume of *P*_*2*_ represents the probability that the patients cooperate in HDTS. These are calculated as


$$V_{P_{1}}=\frac{2\,\left(E_{1}-\lambda \,E_{2}\right)-\left(1-\lambda \right)\,\nu \,\left(\omega \,E_{2}\right)}{2\,\nu^{\prime }\,\left(\theta \,E_{1}\right)}$$

$$V_{P_{2}}=\frac{2\,\lambda \,E_{2}+\left(1-\lambda \right)\,\nu \,\left(\omega \,E_{2}\right)-2\,E_{1}+2\,\nu^{\prime }\,\left(\theta \,E_{1}\right)}{2\,\nu^{\prime }\,\left(\theta \,E_{1}\right)}$$

**Corollary 3:** The probability of patients cooperate in HDTS increases with the increase in the probability that the local government health departments and medical institutions cooperate with the system; at the same time, it increases with the increase of the recovery rate after initial treatment in primary care and the proportion of patients cost savings when participating in referrals; if α = 1, then it increases with the increase of additional medical insurance reimbursement ratio in primary-level hospitals when the ratio of the cost of treatment in primary care to the average cost of treatment in secondary and tertiary hospitals is greater than a certain value, that is *E*_1_/*E*_2_ > λ + (1−λ)ω/2.

### Equilibrium and stability analysis

From the above discussion, it is clear that the dynamic system of the tripartite game in promoting HDTS is as follows:


(11) $$\left\{ \begin{array}{c} F\,\left(x\right)=x\,\left(1-x\right)\,\left[z\,\nu \,\left(\theta \,E_{1}\right)+y\,\nu \,\left(S\right)+\left(1-z\right)\,P\right]\\ F\,\left(y\right)=y\,\left(1-y\right)\,\left[z\,\left(C_{1}-C_{2}\right)+x\,\nu^{\prime }\,\left(S\right)-C_{3}+C_{2}\right]\\ F\,\left(z\right)=z\,\left(1-z\right)\,\left[y\,\left(1-\lambda \right)\,\nu \,\left(\omega \,E_{2}\right)+x\,\nu^{\prime }\,\left(\theta \,E_{1}\right)+\lambda \,E_{2}-E_{1}\right] \end{array}\right.$$

This leads to the pure strategy Nash equilibria of the evolutionary game: *E*_1_(0,0,0), *E*_2_(1,0,0), *E*_3_(0,1,0), *E*_4_(0,0,1), *E*_5_(1,1,0), *E*_6_(1,0,1), *E*_7_(0,1,1), *E*_8_(1,1,1). The Jacobian matrix of the tripartite evolutionary game system is as follows:


$$J=\left[\begin{array}{ccc} J_{1}\; & J_{2}\; & J_{3}\\ J_{4}\; & J_{5}\; & J_{6}\\ J_{7}\; & J_{8}\; & J_{9} \end{array}\right]=\left[\begin{array}{ccc} \partial F\,\left(x\right)\,/\,\partial x & \partial F\,\left(x\right)\,/\,\partial y & \partial F\,\left(x\right)\,/\,\partial z\\ \partial F\,\left(y\right)\,/\,\partial x & \partial F\,\left(y\right)\,/\,\partial y & \partial F\,\left(y\right)\,/\,\partial z\\ \partial F\,\left(z\right)\,/\,\partial x & \partial F\,\left(z\right)\,/\,\partial y & \partial F\,\left(z\right)\,/\,\partial z \end{array}\right]$$

$$=\left[\begin{array}{c} \left(1-2\,x\right)\,\left[z\,\nu \,\left(\theta \,E_{1}\right)+y\,v\,\left(S\right)+\left(1-z\right)\,P\right]\\ y\,\left(1-y\right)\,\nu^{\prime }\,\left(S\right)\\ z\,\left(1-z\right)\,\nu^{\prime }\,\left(\theta \,E_{1}\right) \end{array}\,\,\begin{array}{c} x\,\left(1-x\right)\,\nu \,\left(S\right)\\ \left(1-2\,y\right)\,\left[z\,\left(C_{1}-C_{2}\right)+x\,\nu^{\prime }\,\left(\theta \,E_{1}\right)+\lambda \,E_{2}-E_{1}\right]\\ z\,\left(1-z\right)\,\left(1-\lambda \right)\,\nu \,\left(\omega \,E_{2}\right) \end{array}\,\,\begin{array}{c} x\,\left(1-x\right)\,\left[\nu \,\left(\theta \,E_{1}\right)-P\right]\\ y\,\left(1-y\right)\,\left(C_{1}-C_{2}\right)\\ \left(1-2\,z\right)\,\left[y\,\left(1-\lambda \right)\,\nu \,\left(\omega \,E_{2}\right)+x\,\nu^{\prime }\,\left(S\right)-C_{3}+C_{2}\right] \end{array}\right]$$

From the first method of Lyapunov, let the Jacobian matrix be **A**. If all the eigenvalues of **A** have negative real parts, then the equilibrium is ESS (asymptotic equilibrium); if all the eigenvalues of A contain positive real parts, then the equilibrium is non-stable. The stability analysis of the above eight pure strategy equilibria is thus shown in Table 2.

**TABLE 2:** Stability analysis of equilibria based on Jacobian matrix.

Equilibrium	Jacobian matrix eigenvalues		Stability	Con
	λ_1_, λ_2_, λ_3_	RPS		
*E*_1_(0,0,0)	*P*, *C*_2_−*C*_3_, λ*E*_2_−*E*_1_	(+, +, *U*)	Non-Stable	\
*E*_2_(1,0,0)	−*P*, ν′(*S*) + *C*_2_−*C*_3_, λ*E*_2_−*E*_1_ + ν′(θ*E*_1_)	(−, +, *U*)	Non-Stable	\
*E*_3_(0,1,0)	ν(*S*) + *P*,−*C*_2_ + *C*_3_, λ*E*_2_ + (1−λ)ν(ω*E*_2_)−*E*_1_	(*U*,−,*U*)	*ESS*	a
*E*_4_(0,0,1)	ν(θ*E*_1_),*C*_1_−*C*_3_,−λ*E*_2_ + *E*_1_	(−, +, *U*)	Non-Stable	\
*E*_5_(1,1,0)	$\begin{array}{l} -\nu (S)-P,-\nu (S)-C_{2}+C_{3},\\ (1-\lambda )\nu (\omega E_{2})+\nu '(\theta E_{1})+\lambda E_{2}-E_{1} \end{array}$	(*U*,−,*U*)	*ESS*	b
*E*_6_(1,0,1)	−ν(θ*E*_1_),*S* + *C*_1_−*C*_3_,−λ*E*_2_ + *E*_1_−ν′(θ*E*_1_)	(+, +, *U*)	Non-Stable	\
*E*_7_(0,1,1)	$\begin{array}{l} \nu (\theta E_{1})+\nu (S),-C_{1}+C_{3},\\ -(1-\lambda )v(\omega E_{2})-\lambda E_{2}+E_{1} \end{array}$	(−,−,*U*)	*ESS*	c
*E*_8_(1,1,1)	$\begin{array}{l} -\nu (\theta E_{1})-\nu (S),-\nu '(S)-C_{1}+C_{3},\\ -(1-\lambda )v(\omega E_{2})-v(\theta E_{1})-\lambda E_{2}+E_{1} \end{array}$	(+, −, *U*)	Non-Stable	\

RPS, real part sign; U denotes the uncertainty of whether the real part of the eigenvalue is positive or negative; Con, condition, condition a is −ν(S) > *P, λE*_2_ + (1−*λ*)ν(ω*E*_2_)−*E*_1_ < 0, condition b is −ν(S) < *P*, (1−*λ*)ν(ω*E*_2_) + *ν*′(θ*E*_1_) + *λE*_2_−*E*_1_ < 0, condition c is −(1−*λ*)ν(ω*E*_2_)−*λE*_2_ + *E*_1_ < 0.

**Corollary 4:** The equilibrium is *E*_3_(0,1,0) and the ESS is {D,C,D} when −ν(*S*) > *P*, λ*E*_2_ + (1−λ)ν(ω*E*_2_)−*E*_1_ < 0.

Under this circumstance, the financial cost borne by local government health departments to support HDTS is greater than the penalty they need to pay if they do not support it, so they tend not to be cooperators; since the burden brought to the whole medical system by defecting HDTS is much greater than the additional cost of cooperating, and the local government health departments deploy subsidies, and medical institutions tend to be cooperators to obtain financial support; patients are more inclined not to cooperate with HDTS but to go directly to secondary and tertiary hospitals due to the lower cure rate of primary care institutions.

**Corollary 5:** The equilibrium is *E*_5_(1,1,0) and the ESS is {C,C,D} when −ν(*S*) < *P*, (1−λ)ν(ω*E*_2_) + ν′(θ*E*_1_) + λ*E*_2_−*E*_1_ < 0.

Under this circumstance, the cost for local government health departments to support HDTS is less than the penalty required by higher authorities, so they will tend to be cooperators; medical institutions still tend to cooperate with the referral process to reduce losses because the opportunity cost of not participating in HDTS is too large; patients are still not cooperators because the cure rate in primary care institutions is getting lower.

**Corollary 6:** The equilibrium is *E*_7_(0,1,1) and the ESS is {D,C,C} when −(1−λ)ν(ω*E*_2_)−λ*E*_2_ + *E*_1_ < 0.

Under this circumstance, the recovery rate of patients treated by primary care institutions is significantly higher, so they tend to cooperate in HDTS and are willing to choose primary-level hospitals for the first consultation; both medical institutions and patients becoming cooperators is the goal achieved for local government health departments, for which they can avoid penalties and therefore turn to the strategy of not supporting HDTS.

### Simulation of the ESS

To verify the stabilization of the above three equilibria, the simulation of the tripartite evolutionary game process is performed using MATLAB. According to the medical data collected in a province in southern China, the initial values of the parameters are set as follows to verify **Corollary 4**: *S = 60*, θ = 0.1, *B*_1_ = 100, *L = 10*, *P = 40*, *C*_1_ = 6, *C*_2_ = 10, *C*_3_ = 3, *I*_1_ = 60, *I*_2_ = 30, *E*_1_ = 45, *E*_2_ = 70, λ = 0.3, ω = 0.3, *B*_2_ = 100.

**FIGURE 4:**
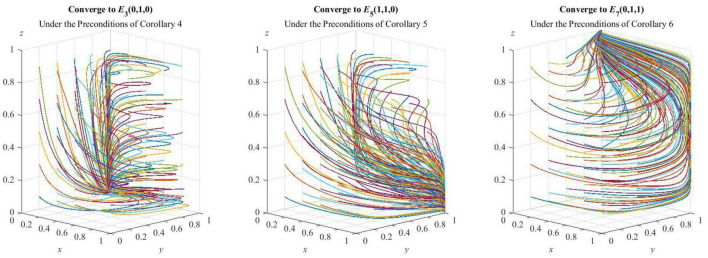
Simulation verification of the ESS.

Here, the units of the non-proportional parameters are unified to 100,000; to verify **Corollary 5**, based on the previous parameter given, let *S = 40* and *P = 80*; to verify **Corollary 6**, based on the previous parameter changes, let *E*_1_ = 30. The fitting results are shown in Figure 4.

As can be seen from Figure 4, under the preconditions of **Corollary 4**, **Corollary 5**, and **Corollary 6**, the three sets of values are, respectively, evolved 50 times from different initial strategy combinations, and the strategy combinations of the tripartite evolutionary game of HDTS finally converge to {D,C,D}, {C,C,D}, and {D,C,C}, thus indicating that the simulation is consistent with the analysis of strategic stability, which has guiding significance for the promotion of HDTS.

### Sensitivity analysis of each parameter

To further explore the influence of each parameter on the whole game system, we assumed the initial probability value of 0.2 as cooperators in HDTS for local government health departments, medical institutions, and patients based on the parameter values consistent with **Corollary 6**, which means that the evolutionary stable strategy combination is *E*_7_(0,1,1). The perturbation added to each parameter value is simulated for sensitivity analysis and as a verification of **Corollary 1**, **Corollary 2**, and **Corollary 3**.

**FIGURE 5:**
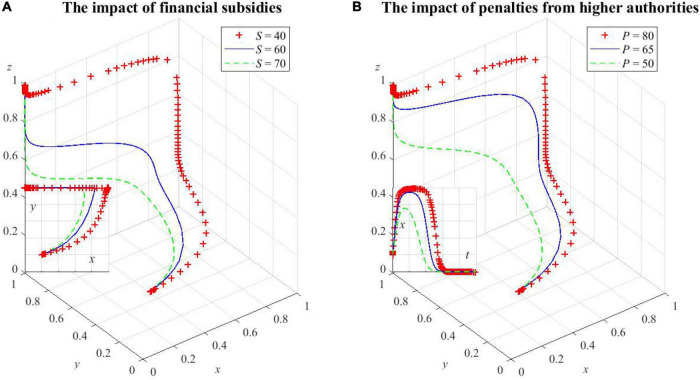
The impact of factors on local government health departments’ decision making. **(A)** The impact of financial subsidies on health departments’ decision making, **(B)** the impact of penalties from higher authorities on health departments’ decision making.

As shown in Figure 5A, the increase in financial subsidies to medical institutions by local government health departments can accelerate the evolution of medical institutions’ active participation in HDTS and also make health departments evolve from cooperators to defectors more quickly. As *S* increases, the probability of medical institutions cooperating with the system increases due to the fact that the increase of subsidies has a greater incentive for them, while the probability of health departments being cooperators decreases due to the increase in financial costs as a result of increased subsidies.

As shown in Figure 5B, increasing penalties *P* can increase the upper limit of the probability of local government health departments being cooperators, which will lead to a longer period of maintaining a high probability of supporting HDTS, and the moment when the probability starts to decline is correspondingly delayed. However, this probability will eventually converge to 0. This is because the social benefits brought by defecting HDTS are not enough to compensate for the losses caused by the increase in penalties. As a result, they tend to be cooperators at this point. After the convergence to the equilibrium, medical institutions and patients actively participate in HDTS. The additional social benefits will increase and the local government health departments will not be punished by higher authorities, so the probability of health departments being cooperators will gradually decrease.

**FIGURE 6:**
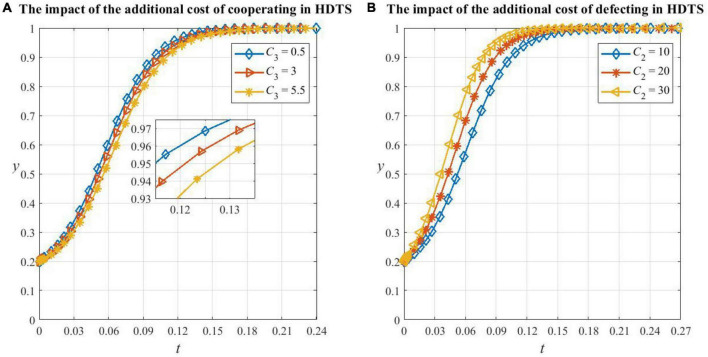
The impact of factors on medical institutions’ decision making. **(A)** The impact of the additional cost of cooperating in HDTS on medical institutions’ decision making, **(B)** the impact of the additional cost of defecting in HDTS on medical institutions’ decision making.

As shown in Figure 6A, the probability of medical institutions cooperating in HDTS decreases and convergence slows as the additional cost of cooperating in HDTS increases. However, when the whole medical system actively participates in the referral process, the operational efficiency increases, and the benefits are much greater than the additional cost of participating in HDTS, so medical institutions will still evolve to being cooperators.

As shown in Figure 6B, the increase in the additional cost of defecting in HDTS will increase the probability of medical institutions cooperating in HDTS. This is because, in this situation, they need to bear the extra cost caused by the low efficiency of medical resource utilization and also cannot obtain government subsidies and extra benefits from participating in HDTS, so the increase of various opportunity costs will motivate medical institutions to be cooperators.

**FIGURE 7:**
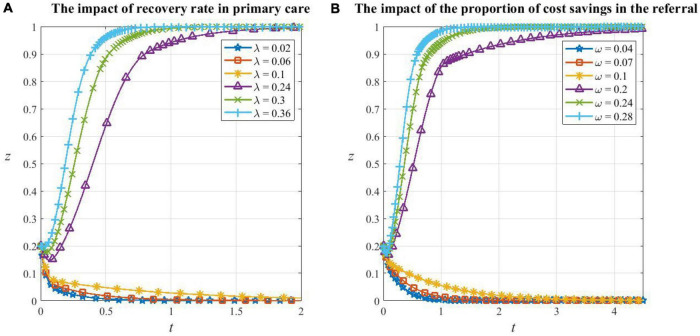
The impact of factors on patients’ decision making. **(A)** The impact of recovery rate in primary care on patients’ decision making, **(B)** the impact of the proportion of cost savings in the referral on patients’ decision making.

From Figures 7A, B, it can be seen that the critical values of recovery rate λ and cost saving ratio ω are between the range 0.1 to 0.24 and 0.1 to 0.2, respectively. When the two parameters are less than their critical values, patients will evolve in the direction of not cooperating in HDTS, and the smaller the parameter value, the smaller the probability of cooperating and the faster it will converge to 0. In this situation, the system has already undergone large changes and the convergence point degenerates to *E*_5_(1,1,0) in **Corollary 5**. This is because if the cure rate of primary care is too low and the patient’s condition has a higher risk of deterioration, which increases the potential medical expense, then the patient will be more willing to consult secondary or tertiary hospitals which are equipped with better medical resources; if the savings, when the patient participates in the referral, are too small to compensate for the opportunity cost brought by cooperating in HDTS, then the patient will also choose a higher-level hospital. Meanwhile, we found that the convergence speed of patients’ strategy evolution when λ is changed is greater than that when ω is changed, which indicates that the recovery rate is a more important factor in patients’ strategy choice than the treatment cost.

**FIGURE 8:**
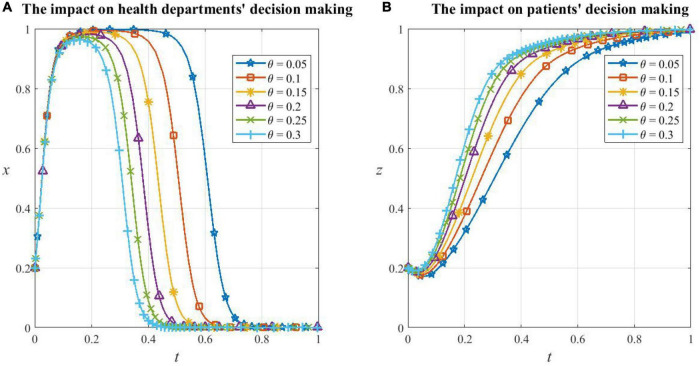
The impact of additional health insurance reimbursement rate. **(A)** The impact of additional health insurance reimbursement rate on local government health departments’ decision making, **(B)** the impact of additional health insurance reimbursement rate on patients’ decision making.

From Figures 8A, B, we can see that the increase in the additional reimbursement rate of primary care θ will accelerate the evolution of local government health departments from active support to inactive support and also the evolution of patients’ cooperation in HDTS. With the increase θ, the probability of health departments choosing to support HDTS increases at about the same rate, but the phase to maintain the high probability is shortened and soon enters the decreasing stage. This is because the increase in the health insurance reimbursement rate in primary care makes patients more willing to cooperate in HDTS, which can bring social benefits and allow the system to converge to equilibrium quickly. Then, the local government health departments will ease up on the support of HDTS due to the need for financial expenditure reduction and the fact that they will not be punished by higher authorities since the aim of HDTS is achieved.

### The effect of psychological perceived value on the system

It is measured that when α = 0.88 and β = 2.25, the value function can represent the approximate behavioral preferences of any decision-maker (Li et al., 2022). We can rewrite the replicator dynamic equations in Eq. 11 as ones with α and β.


(12) $$\left\{ \begin{array}{c} F\,\left(x\right)=x\,\left(1-x\right)\,\left[-z\,\beta \,{\left(\theta \,E_{1}\right)}^{\alpha }-y\,\beta \,S^{\alpha }+\left(1-z\right)\,P\right]\\ F\,\left(y\right)=y\,\left(1-y\right)\,\left[z\,\left(C_{1}-C_{2}\right)+x\,S^{\alpha }-C_{3}+C_{2}\right]\\ F\,\left(z\right)=z\,\left(1-z\right)\,\left[y\,\left(1-\lambda \right)\,{\left(\omega \,E_{2}\right)}^{\alpha }+x\,{\left(\theta \,E_{1}\right)}^{\alpha }+\lambda \,E_{2}-E_{1}\right] \end{array}\right.$$

To investigate the effect of the risk sensitivity coefficient α and the loss avoidance factor β on the system evolution, the values of 0.5, 0.7, 0.88, and 1 are taken for α and 1, 1.5, 2.25, and 3 for β, respectively. The game system is still based on the parameter values consistent with **Corollary 6,** and let the initial values of *x*, *y*, and *z* all be 0.2. With these preconditions, the sensitivity of the strategy choice of each participant to α and β is simulated as follows.

(1) Sensitivity analysis of game subjects to α

**FIGURE 9:**
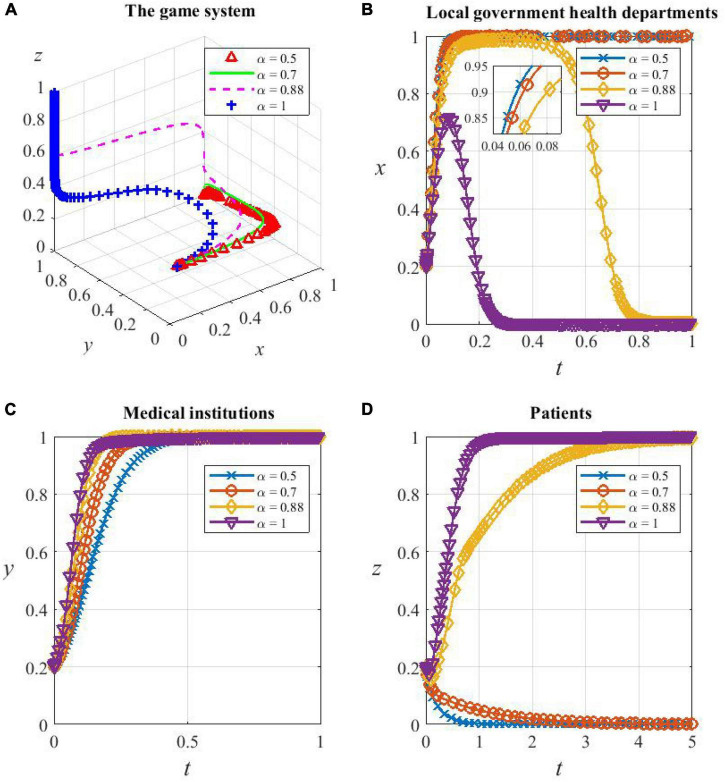
The game subjects’ sensitivity to α. **(A)** The whole game system’s sensitivity to α, **(B)** local government health departments’ sensitivity to α, **(C)** medical institutions’ sensitivity to α, and **(D)** patients’ sensitivity to α.

From Figure 9A, it can be seen that the game system is very sensitive to the risk sensitivity coefficient α, and changes in α cause the equilibrium of the system to change. When α = 0.5 and 0.7, the system evolves toward the strategy combination {C,C,D} and eventually stabilizes, and the smaller the value α, the faster it evolves; when α = 0.88 and 1, the system evolves toward the strategy combination {D,C,C} and eventually stabilizes, and the larger the value of α, the faster it evolves.

As can be seen from Figures 9B–D, when α = 0.5 and 0.7, which means the level of the risk sensitivity coefficient is low, the perception of health departments’ subsidies ν(*S*) and additional reimbursement ν(θ*E*_1_) is faint. They perceive the cost of supporting HDTS to be small and therefore evolve toward being cooperators. Medical institutions have little perception of the subsidies ν′(*S*) received, and the incentive effect of subsidies is not great, so the evolution to cooperate in HDTS is slowed down. Patients’ perception of additional reimbursement ν′(θ*E*_1_) and referral savings ν(ω*E*_2_) is small, so they do not perceive significant benefits from participating in the system, so they will evolve in the direction of being defectors.

When α exceeds a certain threshold (0.5–0.88), which means that the risk sensitivity coefficient is at a high level, for example, α increases from 0.88 to 1, the degree of increase in the probability of them being cooperators and the duration of maintaining the high probability decreases sharply, leading to the acceleration of the evolution of finally inactive support. This is because health departments are more inclined to reduce the support as soon as possible because of the perceived increased cost of promoting HDTS, while medical institutions and patients are more inclined to support it because the perceived benefits are more significant.

Overall, the evolutionary tendency of the game system toward the optimal strategy combination {D,C,C} will strengthen as α increases, but α being too large also makes the willingness of health departments to support HDTS drop significantly, and therefore, the dividend period of support provided for medical institutions and patients becomes shorter.

(2) Sensitivity analysis of game subjects to β

**FIGURE 10:**
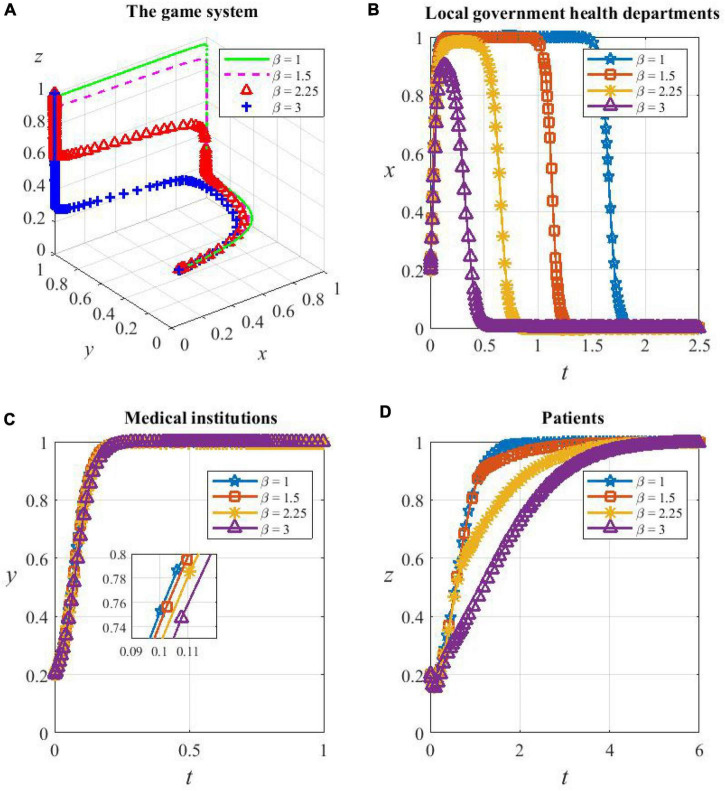
**(A)** The game subjects’ sensitivity to β. The whole game system’s sensitivity to β, **(B)** local government health departments’ sensitivity to β, **(C)** medical institutions’ sensitivity to β, and **(D)** patients’ sensitivity to β.

From Figure 10A, the game system is sensitive to the loss avoidance factor β. However, changes in β do not change the equilibrium of the system, and they only have an impact on the speed of convergence. The system evolves toward and eventually stabilizes at the strategy combination {D,C,C} no matter what level β is on, and the larger the value β, the slower the system converges.

As shown in Figures 10B–D, the higher the value β, the faster the health department evolves from cooperating in HDTS to not supporting it; the speed of medical institutions evolving to be cooperators decreases, but the magnitude is very small; the speed of patients evolving to cooperate becomes very slow, which is the determinant of the slow convergence of the system. This is because, as β increases, health departments become more aware of their loss when supporting HDTS, which strengthens the tendency of them to be defectors and prematurely stop providing additional reimbursements to patients who consult primary care institutions. At the same time, the motivation of patients to cooperate depends mostly on the recovery rate in primary care institutions and the perceived benefits of cost savings when participating in referrals. The improvement of these two factors requires higher medical standards and higher operational efficiency of the health care system, which will take a long time to achieve in reality, and therefore, the probability of patients’ cooperation in HDTS converges to one more slowly.

Overall, as β increases, the game system evolves more slowly toward the optimal strategy combination {D,C,C}. The sensitivity of patients with β is mainly influenced by local government health departments and medical institutions.

## Further discussion

### Reasons for using stochastic evolutionary game model

In our process of establishing the above model, the introduction of prospect theory shows the internal influence of psychological factors on HDTS (Linder et al., 2021), which only reflects the limited rationality of subjects inside this game system. In reality, the uncertainty of external factors also has a significant impact on the implementation of HDTS (Sun and Gu, 2022). Specifically, changes in the operating environment of the medical system, unexpected personal situations of patients, and interference from other external factors are stochastic; therefore, consideration of stochastic factors can improve the accuracy of models (Wang et al., 2022). Based on this, some scholars regard the strategy evolution process as a random jump between multiple states and use the traditional game to construct a stochastic game model, but complete rationality becomes the biggest constraint (Rand et al., 2013). To improve the validity of the model, this study draws on the concept of Gaussian white noise to construct a stochastic evolutionary game model under asymmetric conditions for describing the real-time stochastic dynamic evolutionary process of the promotion of HDTS. The Itô stochastic differential equation of the three-party game is solved numerically, and the stability analysis of the strategy selection states of the three parties is carried out according to the stability discriminant theorem of the stochastic differential equation.

### Stochastic evolutionary game modeling

To facilitate the study of the influence of external interference on the decision of three parties, we assume *α* = *β* = 1. It is important to note that the fraction of defectors in local government health departments is remarked with *x*, while the fraction of cooperators is 1 − *x*. The same goes for medical institutions and patients. Since *x*, *y*, *z*∈[0,1], 1 − *x*, 1 − *y*, 1 − *z* are non-negative and have no effect on the final strategy choice, so the previous replicator dynamic equations are modified as follows:


(13) $$\left\{ \begin{array}{c} d\,x\,\left(t\right)=\left[-\left(P+\theta \,E_{1}\right)\,z-y\,S+\theta \,E_{1}+S\right]\,x\,\left(t\right)\,d\,t\\ d\,y\,\left(t\right)=\left[\left(C_{1}-C_{2}\right)\,z+S\,x-C_{1}+C_{3}-S\right]\,y\,\left(t\right)\,d\,t\\ dz\left(t\right)=[\left(1-\lambda \right)\omega E_{2}y+\theta E_{1}x-\left(1-\lambda \right)\omega E_{2}\\ -\lambda E_{2}+\left(1-\theta \right)E_{1}]z(t)dt \end{array}\right.$$

Hence, by drawing on the concept of Gaussian white noise, the stochastic differential equation is used to describe the various types of random disturbance existing in the game system, and the stochastic replicator dynamic differential equation for each participant can be obtained as follows:


(14) $$\left\{ \begin{array}{c} d\,x\,\left(t\right)=\left[-\left(P+\theta \,E_{1}\right)\,z-y\,S+\theta \,E_{1}+S\right]\,x\,\left(t\right)\,d\,t\\ +\delta \,\sqrt{\left(1-x\,\left(t\right)\right)\,x\,\left(t\right)}\,d\,\omega \,\left(t\right)\\ d\,y\,\left(t\right)=\left[\left(C_{1}-C_{2}\right)\,z+S\,x-C_{1}+C_{3}-S\right]\,y\,\left(t\right)\,d\,t\\ +\delta \,\sqrt{\left(1-y\,\left(t\right)\right)\,y\,\left(t\right)}\,d\,\omega \,\left(t\right)\\ dz\left(t\right)=[\left(1-\lambda \right)\omega E_{2}y+\theta E_{1}x-\left(1-\lambda \right)\omega E_{2}-\lambda E_{2}\\ +\left(1-\theta \right)E_{1}]z(t)dt+\delta \sqrt{\left(1-z\,\left(t\right)\right)\,z\,\left(t\right)}d\omega (t) \end{array}\right.$$

ω(*t*) is the standard one-dimensional Brownian motion process with increments Δω(*t*) = ω(*t* + *h*)−ω(*t*) obeying the normal distribution $N\,\left(0,\sqrt{h}\right)$, and the meaning of the second half of the stochastic differential equation is illustrated using the first equation in Eq. 14 as an example:

1. Many perturbations are affecting the tripartite participation in HDTS, both internal and external, and each factor does not play a decisive role, which illustrates the significance of normal distribution.
2. $\delta \,\sqrt{\left(1-x\,\left(t\right)\right)\,x\,\left(t\right)}\,d\,\omega \,\left(t\right)$ is the stochastic disturbance, and (1−*x*(*t*))*x*(*t*) reaches its maximum only at 1−*x*(*t*) = *x*(*t*), which is in line with the actual situation where the probability of two decisions differs greatly and people tend to choose the strategy with the higher probability due to the herd mentality. If the two probabilities differ little, people often waver and the decision is easily disturbed by external factors.
3. δ indicates the intensity coefficient of stochastic disturbance, and the coefficient size is related to the medical culture. In urban areas, HDTS is less likely to be obstructed, since there exists less interference intensity in various aspects because of the well-constructed medical environment, adequate policy propaganda, high quality of the medical population, strong willingness to cooperate, and more participation in unit medical checkups (Peen et al., 2010). In rural areas, the lack of medical resources, the difficulty of seeking medical treatment, the severe aging of the rural population, the recurrence of epidemics in recent years, and the tendency to avoid medical treatment due to economic level and traditional concepts all have caused greater interference in the implementation of HDTS (Parks et al., 2003). In summary, the urbanization level is inversely correlated with the intensity coefficient of stochastic disturbance.

### Stability analysis of the equilibrium solution

The Itô stochastic differential equation is expressed as


(15) d⁢x⁢(t)=f⁢(t,x⁢(t))⁢d⁢t+g⁢(t,x⁢(t))⁢d⁢ω⁢(t)

According to the stability discriminant theorem for stochastic differential equations (Ajmone Marsan et al., 2016), there exists a positive, continuous function *V*(*t*, *x*) with positive constants *c*_*1*_, *c*_*2*_, such that *c*_1_ |*x*|*^p^* ≤ *V*(*t*, *x*) ≤ *c*_2_ |*x*|*^p^*, *t* ≥ 0.

1. If there exists a positive constant γ, such that *LV*(*t*, *x*) ≤ − γ*V*(*t*, *x*), *t* ≥ 0, then it implies that the global exponential stability in *p*th mean of Eq. 15 and *E*|*x*(*t*, *x*_0_)|*^p^* ≤ (*c*_2_/*c*_1_) |*x*_0_|*^p^ e*^−γ^ *^t^*, *t* ≥ 0.
2. If there exists a positive constant γ, such that *LV*(*t*, *x*) ≥ γ *V*(*t*, *x*), *t* ≥ 0, then it implies the global exponential instability in *p*th mean of Eq. 15 and *E*|*x*(*t*, *x*_0_)|*^p^* ≥ (*c*_2_/*c*_1_) |*x*_0_|*^p^ e*^−γ^*^t^*, *t* ≥ 0.

For Eq. 14, Suppose that *V*_*t*_(*t*, *x*) = *x*, *V*_*t*_(*t*, *y*) = *y*, *V*_*t*_(*t*, *z*) = *z*, *c*_1_ = *c*_2_ = 1, *p = 1*, γ = 1, we obtain:


(16) $$\left\{ \begin{array}{c} L\,V\,\left(t,x\right)=f\,\left(t,x\right)=x\,\left[-\left(P+\theta \,E_{1}\right)\,z-y\,S+\theta \,E_{1}+S\right]\\ L\,V\,\left(t,y\right)=f\,\left(t,y\right)=y\,\left[\left(C_{1}-C_{2}\right)\,z+S\,x-C_{1}+C_{3}-S\right]\\ LV\left(t,z\right)=f\left(t,z\right)=z[\left(1-\lambda \right)\omega E_{2}y+\theta E_{1}x\\ -\left(1-\lambda \right)\omega E_{2}-\lambda E_{2}+\left(1-\theta \right)E_{1}] \end{array}\right.$$

Therefore, if there is the zero-solution exponential stability of Eq. 16, then


(17) $$\left\{ \begin{array}{c} x\,\left[-\left(P+\theta \,E_{1}\right)\,z-y\,S+\theta \,E_{1}+S\right]\leq -x\\ y\,\left[\left(C_{1}-C_{2}\right)\,z+S\,x-C_{1}+C_{3}-S\right]\leq -y\\ z[\left(1-\lambda \right)\omega E_{2}y+\theta E_{1}x-\left(1-\lambda \right)\omega E_{2}-\lambda E_{2}\\ +\left(1-\theta \right)E_{1}]\leq -z \end{array}\right.$$

Accordingly, if there is the zero-solution exponential instability of Eq. 16, then:


(18) $$\left\{ \begin{array}{c} x\,\left[-\left(P+\theta \,E_{1}\right)\,z-y\,S+\theta \,E_{1}+S\right]\geq x\\ y\,\left[\left(C_{1}-C_{2}\right)\,z+S\,x-C_{1}+C_{3}-S\right]\geq y\\ z[\left(1-\lambda \right)\omega E_{2}y+\theta E_{1}x-\left(1-\lambda \right)\omega E_{2}-\lambda E_{2}\\ +\left(1-\theta \right)E_{1}]\geq z \end{array}\right.$$

### Numerical solution of stochastic differential equations

Since the non-linear Itô stochastic differential equation cannot be solved analytically, we used the Stochastic Taylor Expansion and the Itô differential formulation method for the stochastic numerical approximation. For Eq. 15, let *t* ∈ [*t*_0_, *T*], *x*(*t*_0_) = *x*_0_, *x*_0_ ∈ *R*. *d*ω(*t*) obeys the normal distribution *N*(0, Δ*t*). Let *h* = (*T*−*t*_0_)/*N*, *t*_*n*_ = *t*_0_ + *nh*, *t*_0_ < *t*_1_ < … < *t*_*n*_ < … < *t*_*N*_ = *T*. We can find the numeric solution for stochastic differential equations using the explicit Milstein method of Eq. 19 as follows (Gobet et al., 2005):


(19) $$x\,\left(t_{n+1}\right)=x\,\left(t_{n}\right)+h_{n}\,f\,\left(x\,\left(t_{n}\right)\right)+\Delta \,\omega_{n}\,g\,\left(x\,\left(t_{n}\right)\right)+\frac{1}{2}\,\left[{\left(\Delta \,\omega_{n}\right)}^{2}-h_{n}\right]\,g\,\left(x\,\left(t_{n}\right)\right)\,g^{\prime }\,\left(x\,\left(t_{n}\right)\right)$$

In Eq. 19, the step size is *h*_*n*_ = *t*_*n* + 1_−*t*_*n*_, Δω_*n*_ = ω(*t*_*n* + 1_)−ω(*t*_*n*_)∼*N*(0, *h*_*n*_), ω(*t*_0_) = 0.

The form of numeric solutions can be obtained according to the above expanding method for Eq. 14:


(20) $$\left\{ \begin{array}{c} x\,\left(t_{n+1}\right)=x\,\left(t_{n}\right)+h_{n}\,\left[-\left(P+\theta \,E_{1}\right)\,z\,\left(t_{n}\right)-S\,y\,\left(t_{n}\right)+\theta \,E_{1}+S\right]\\ +\Delta \,\omega_{n}\,\delta \,\sqrt{\left(1-x\,\left(t_{n}\right)\right)\,x\,\left(t_{n}\right)}+\frac{1}{4}\,\delta^{2}\,\left[{\left(\Delta \,\omega_{n}\right)}^{2}-h_{n}\right]\\ \left(1-2\,x\,\left(t_{n}\right)\right)\\ y\left(t_{n+1}\right)=y\left(t_{n}\right)+h_{n}[\left(C_{1}-C_{2}\right)z\left(t_{n}\right)+Sx\left(t_{n}\right)-C_{1}\\ +C_{3}-S]\\ +\Delta \,\omega_{n}\,\delta \,\sqrt{\left(1-y\,\left(t_{n}\right)\right)\,y\,\left(t_{n}\right)}+\frac{1}{4}\,\delta^{2}\,\left[{\left(\Delta \,\omega_{n}\right)}^{2}-h_{n}\right]\\ \left(1-2\,y\,\left(t_{n}\right)\right)\\ z\left(t_{n+1}\right)=z\left(t_{n}\right)+h_{n}[\left(1-\lambda \right)\omega E_{2}y\left(t_{n}\right)+\theta E_{1}x\left(t_{n}\right)\\ -\left(1-\lambda \right)\omega E_{2}-\lambda E_{2}+\left(1-\theta \right)E_{1}]\\ +\Delta \,\omega_{n}\,\delta \,\sqrt{\left(1-z\,\left(t_{n}\right)\right)\,z\,\left(t_{n}\right)}+\frac{1}{4}\,\delta^{2}\,\left[{\left(\Delta \,\omega_{n}\right)}^{2}-h_{n}\right]\\ \left(1-2\,z\,\left(t_{n}\right)\right) \end{array}\right.$$

### Impact of medical cultures on the game system

In the simulation, the step size *h*_*n*_ = 0.001. The initial state is assumed as *x*(0) = *y*(0) = *z*(0) = 0.5 and the parameter values are consistent with **Corollary 6**, that is, consistent with the equilibrium {D,C,C}. It can be confirmed theoretically that, with the given parameter values, the proportion of defectors in medical institutions and patients has zero-solution exponential stability (Eq. 17) and the proportion of defectors in health departments does not (Eq. 18).

The Milstein method (Eq. 20) is used for numerical simulation, and the values of 1.5, 3, and 5 are taken for the intensity coefficient of stochastic disturbance δ. From the previous analysis, it is known that the larger value represents the lower degree of urbanization, and the above three values can represent the degree of disturbance in the promotion of HDTS under the medical cultures of urban, town, and rural, respectively.

**FIGURE 11:**
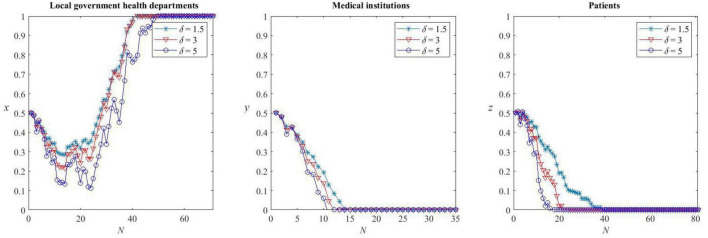
Stochastic evolution of strategies under different medical cultures.

As can be seen from Figure 11, the evolution process shows a certain volatility, indicating that the system evolution is disturbed by stochastic factors. Different intensity coefficients of stochastic disturbance have different effects on the evolution rate of the game system. The strategy evolution of the local government health departments is the most sensitive to the perturbation changes. The inadequate construction of the rural healthcare environment motivates the local health departments to actively support HDTS for a longer time and converge to the non-zero solution for a longer time compared with urban and town areas.

The strategy evolution of medical institutions is the least sensitive to the perturbation changes, and the rate of convergence to the zero solution is roughly the same for all medical cultures. This is related to the higher opportunity cost of being defectors in HDTS.

The strategy evolution of patients is moderately sensitive to the perturbation changes, and the number of simulations required for patients’ strategies to converge to the cooperation state is less in rural areas than in urban areas, that is, patients in rural areas are more willing to cooperate in HDTS. This is because, in the evolutionary game model constructed above, the interference of health departments has become an important factor that affects the cooperation of patients. The purpose of HDTS is to make patients comply with the rules of the first consultancy in primary care and orderly referral. Therefore, the unstable medical environment in rural areas requires continuous follow-up of the subsidy policy of health departments and creates adequate benefits for rural patients so that they show active cooperation with the system. It is also clear from Figure 11 that the more the support of HDTS of health departments under the rural medical culture, the more quickly rural patients will become cooperators.

## Conclusion and recommendations

### Conclusion

This study analyzes the evolutionary game among local government health departments, medical institutions, and patients in the context of promoting the HDTS and planning the layout of medical resources, our research found the following:

The probability of local government health departments cooperating in HDTS is negatively correlated with the number of subsidies and additional medical insurance reimbursement for patients, positively correlated with penalties from higher-level authorities, and negatively correlated with the probability of the other two parties being cooperators.

The probability of medical institutions cooperating in HDTS is positively correlated with the subsidies received and the costs borne by being defectors in the system, negatively correlated with the additional costs borne by being cooperators in HDTS, and positively correlated with the probability of the other two parties being cooperators.

The probability of patients cooperating in HDTS is positively correlated with the recovery rate of treatment in primary medical institutions, the additional reimbursement received, and the proportion of cost savings when participating in referrals and positively correlated with the probability of the other two parties being cooperators.

Changes in the risk sensitivity coefficient α alter the equilibrium of the tripartite game system. When α is below a certain threshold (0.7-0.88), the strategic combination of the tripartite game evolves to {C,C,D}. When α is above this threshold, the strategy combination evolves to {D,C,C}. In addition, when α is at a high level, local government health departments perceive that the cost of supporting HDTS increases, so they are inclined to be defectors. However, medical institutions and patients tend to cooperators due to the perceived increase in benefits.

Changes in the loss avoidance factor β do not alter the equilibrium but have a significant impact on the speed of convergence. As β increases, the evolution of the strategy of local government health departments, from a high probability of support of HDTS to eventual non-support, becomes faster; the evolution of medical institutions toward participation in HDTS mildly decreases; the evolution of patients toward cooperation with HDTS slows down significantly. This is because the increase in the loss aversion makes the financial subsidies and additional reimbursement given by local government health departments reduce, which will slow down the speed of improvement in primary care institutions on the one hand, and decrease the recovery rate in primary care and the benefits patients acquire in referral on the other hand.

Different intensity coefficients of stochastic disturbance δ have different effects on the speed of evolution of each game subject in HDTS. Compared with medical cultures in areas with a higher degree of urbanization, the health departments under rural medical culture support HDTS for a longer time. Meanwhile, with the government’s intervention, rural patients are more motivated to cooperate in HDTS, and the system will converge to equilibrium faster.

### Recommendations

Local government health departments need to actively play a regulatory role in the medical services market. First, the local health commission can control the financial support by keeping it within certain limits. While increasing financial input, improving the efficiency of healthcare financial expenditures and avoiding financial waste are also important. Second, local health insurance departments can use health insurance reimbursement policies to play a leveraging role in the formation of a rational order for residents to seek medical treatment. By adjusting health insurance reimbursement ratios, the gap between the reimbursement ratios of medical expenses can be appropriately widened when residents choose different levels of medical institutions for their first consultation. In this way, the government health departments can provide effective guidance for patients in the rural medical culture. At the same time, reasonably set the prices for different levels of hospital visits in the referral system. This will encourage residents to form the habit of seeking medical treatment in community-level institutions for minor illnesses and in the Grade III hospitals for major illnesses, thus promoting the efficient operation of the medical system, and improve the service capacity, treatment level, and equipment configuration of primary care institutions by injecting more resources and letting the treatment quality of primary care hospitals be the same as the secondary and tertiary hospitals, thus improving patients’ satisfaction. Finally, by increasing the publicity of HDTS, we can strengthen the popularization and guidance of the policy so as to improve the residents’ willingness to seek primary care and gradually form a scientific and reasonable order of the medical system. In addition, the National Health Care Commission and the National Health Insurance Bureau are supposed to inspect the local medical environment regularly, strengthen the refined supervision of health insurance funds, promote the standardized use of medical insurance funds by relying on information technology, resolutely prevent insurance frauds from disrupting the medical service market, and create a new regulatory pattern of “health insurance plus credit”.

As providers of medical and health services, medical institutions at all levels should, first of all, take effective measures to improve patients’ sense of fulfillment and trust by improving the level of treatment, service quality, and referral efficiency. It is also advisable to enhance the internal operation governance of medical associations, smooth referral channels, and promote technology sharing among all hospitals. Second, they should clearly define their functional positioning and business division to form a medical service network without conflict of interest and horizontal competition and ensure two-way treatment without barriers. Grade III hospitals should focus on the leading role in medical science, technological innovation, and talent training, gradually reduce the pressure caused by general outpatient clinics with clear diagnoses and stable conditions, and divert patients with chronic diseases. Primary medical institutions should pay more attention to refining internal management processes, improving service capabilities, and creating a comfortable medical environment so as to attract more patients to choose primary care. Finally, the rights and obligations of medical institutions at all levels and the criteria for referrals should be clearly defined, leading to a more convenient referral process for patients to take part in.

Patients, as the demand side of medical services, first, should establish awareness of keeping in good health and have a basic understanding of the disease spectrum, as well as knowledge of the types and prevention measures of common and frequently-occurring diseases, which is of great significance to build a more effective and scientific medical culture. Second, they should change the notion of seeking medical treatment in high-level hospitals for every disease and be confident in the precise management of government health departments and the standardized treatment of medical institutions, especially primary medical institutions. Finally, they should also take the initiative to understand the health policies such as medical insurance reimbursement and referral rules and then actively participate in the process of promoting HDTS in an orderly manner.

### Limitations and future directions

This study explores the decision-making process of the three parties involved in the promotion of the diagnosis and treatment system, which can provide insights for further improvement. Inevitably, there are some limitations to be solved. First, the data and parameter values used in the simulation may not fit the real situation well and can only give the optimal explanation within a certain range. Second, the promotion of the hierarchical medical system also involves the participation of pharmaceutical manufacturing companies, and our study only analyzes the problem from a macroscopic perspective. This could be considered in the follow-up research.

## Data availability statement

The original contributions presented in this study are included in the article/supplementary material, further inquiries can be directed to the corresponding author.

## Author contributions

CT contributed to the model building as well as the manuscript writing. XC contributed to the analysis of the results and made multiple revisions for the final publication. WZ and ZZ supported the total work of CT and contributed to the empirical analysis and text writing. RT, RD, and QX contributed to the overall quality of literature organization and manuscript revision. All authors discussed the conclusions and approved the submitted version.
